# Multi-epitope vaccine design for hepatitis E virus based on protein ORF2 and ORF3

**DOI:** 10.3389/fmicb.2024.1372069

**Published:** 2024-03-21

**Authors:** Qiong Lu, Hao Wu, Jing Meng, Jiangyuan Wang, Jiajing Wu, Shuo Liu, Jincheng Tong, Jianhui Nie, Weijin Huang

**Affiliations:** ^1^Division of HIV/AIDS and Sex-transmitted Virus Vaccines, Institute for Biological Product Control, National Institutes for Food and Drug Control and WHO Collaborating Center for Standardization and Evaluation of Biologicals, Beijing, China; ^2^Wuhan Institute of Biological Products Co., Ltd., Wuhan, China; ^3^State Key Laboratory of Common Mechanism Research for Major Diseases, Suzhou Institute of Systems Medicine, Chinese Academy of Medical Sciences and Peking Union Medical College, Suzhou, Jiangsu, China; ^4^Guangzhou National Laboratory, Guangzhou, China; ^5^Research and Development Department, Beijing Yunling Biotechnology Co., Ltd., Beijing, China; ^6^Changping Laboratory, Beijing, China; ^7^Graduate School of Peking Union Medical College, Beijing, China

**Keywords:** multi-epitope, vaccine, HEV, ORF2, ORF3

## Abstract

**Introduction:**

Hepatitis E virus (HEV), with heightened virulence in immunocompromised individuals and pregnant women, is a pervasive threat in developing countries. A globaly available vaccine against HEV is currently lacking.

**Methods:**

We designed a multi-epitope vaccine based on protein ORF2 and ORF3 of HEV using immunoinformatics.

**Results:**

The vaccine comprised 23 nontoxic, nonallergenic, soluble peptides. The stability of the docked peptide vaccine-TLR3 complex was validated by molecular dynamic simulations. The induction of effective cellular and humoral immune responses by the multi-peptide vaccine was verified by simulated immunization.

**Discussion:**

These findings provide a foundation for future HEV vaccine studies.

## 1 Introduction

Hepatitis E virus (HEV) causes acute viral hepatitis and is spread mainly by fecal–oral transmission (Dalton et al., 2008; Nan and Zhang, 2016). It is self-limiting or asymptomatic in healthy individuals, but causes high mortality in immunocompromised patients, such as pregnant women. The case fatality rate in pregnant women is up to 30% (Qian et al., 2023). There are eight genotypes of *Orthohepevirus A* species in the Hepeviridae family, of which four genotypes infect humans (Marion et al., 2020). Genotypes 1 and 2 exclusively infect humans, whereas genotypes 3 and 4 infect not only humans, but also animals, including swine. Therefore, they are considered zoonotic pathogens and have wide host tropism (Smith et al., 2014; Songtanin et al., 2023).

Hepatitis E virus (HEV) is a 7.2-kb positive-strand RNA virus belonging to the family Hepeviridae, and has three open reading frames (ORFs): ORF1, ORF2, and ORF3 (Smith et al., 2014). ORF1 encodes a large nonstructural polyprotein responsible for HEV replication and transcription. ORF2 is a capsid protein with approximately 660 amino acid residues, and most neutralizing sites are located on its surface. Generally, there are two forms of the ORF2 protein, namely intracellular ORF2 and secreted ORF2 (Montpellier et al., 2018; Yin et al., 2018). HEV is a quasi-enveloped hepatovirus, with the membrane supplied by the host ESCRT system. The membrane protects the virus from the neutralizing antibodies of its host (Feng et al., 2014). ORF3 conducts an ion channel function with a hydrophobic sequence, which is considered a viroporin. ORF3 encodes a protein containing 112 amino acids that is presented on the surface of enveloped HEV (Takahashi et al., 2008). The phosphorylated form of the ORF3 protein interacts with the non-glycosylated form of ORF2 (Tyagi et al., 2002). The a motif containing amino acid proline, serine, alanine, proline (PSAP) motif of ORF3 is necessary for the release of the virions from infected cells (Yamada et al., 2009; Ahmad et al., 2011; Ding et al., 2017). Previous studies have demonstrated that ORF3 not only enhances the production of interferon (IFN), but is also involved in the downregulation of the Toll-like receptor 3 (TLR3) and TLR7 downstream signaling pathways (He et al., 2016; Lei et al., 2018). ORF4, which was discovered in genotype 1, is responsible for the enhancement of viral replication of genotype 1 under endoplasmic reticulum (ER) stress, and this was verified by exogenous introduction of ORF4 into genotype 3 virus (Nair et al., 2016; Yadav et al., 2021).

Two existing hepatitis E vaccines have been evaluated in clinical trials. The Hecolin^®^ vaccine antigen contains amino acids 368–606 of the ORF protein (pORF2) of HEV genotype 1, expressed in *Escherichia coli*. It can provide protection against HEV for approximately 4.5 years (Zhu et al., 2010; Zhang et al., 2015). The recombinant HEV vaccine (rHEV) produced by GlaxoSmithKline (GSK) contains amino acids 112–607 of the ORF2 protein of HEV genotype 1, expressed in baculovirus (Li et al., 2015).

Because the virus is quasi-enveloped, an antibody targeting ORF2 will not fully detect the enveloped virus in cell culture or serum, unless the virus is treated with sufficient detergent and protease (Takahashi et al., 2008). It has been reported that an ORF3 antibody can neutralize this enveloped virus to some extent, but not the virus in feces (Takahashi et al., 2008). A bacterially expressed ORF3 peptide effectively reduced the viral titer, reduced the duration of viremia and fecal shedding, and even partly prevented experimental hepatitis induced with two HEV genotypes: genotype 1 and 4 (Ma et al., 2009). Upon immunization with the bacterially expressed ORF3 protein of strains VaHEV from laying hens and YT-aHEV from broilers, the nucleic acid of avian HEV detected in cloacal swabs vanished within 7 days of infection. This suggests that the immunized antibodies inhibit the proliferation of the virus and provide protection against infection (Syed et al., 2017). HEV ORF3 equipped with the myotropic adeno-associated virus vector (AAVMYO3) induced dose-dependent ORF3 antibodies in mice and the serum immunized by ORF3 inhibited infection by enveloped HEV *in vivo* by 50% compared with that of the control (Maurer et al., 2022). Humans infected with HEV *via* blood transfusion have been reported in many countries (Takahashi et al., 2010).

In regard to the protective roles of ORF3 for enveloped HEV, we first introduce ORF3 as a candidate for multi-epitopic vaccine design.

Immunoinformatic methods have been successfully applied to multiple types of vaccines targeting different pathogens, including HPV, PEDV, *Mycobacterium tuberculosis*, SARS-CoV-2, and HEV (Behmard et al., 2020; Samavia et al., 2022; Arega et al., 2023; Hou et al., 2023; Ikram et al., 2023; Tîrziu et al., 2023). Multi-epitope vaccines designed with this method will stimulate the CD4^+^, CD8^+^ T-cell response and the B-cell response.

In this study, we screened peptides from the ORF2 and ORF3 proteins that stimulated T- and B-cell responses. We also assessed various aspects of the polypeptide vaccine, including its immunogenicity, allergenicity, and physiochemical properties. This study should provide a universal vaccine targeting ORF2 and ORF3 that will eliminate both enveloped and non-enveloped HEV.

## 2 Materials and methods

**FIGURE 1:**
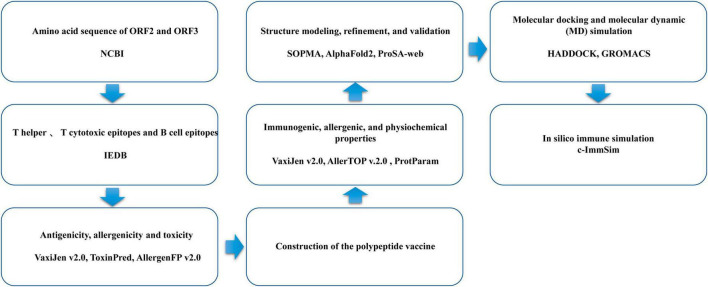
Flowchart of the design of the multi-epitope vaccine.

The flowchart for the design of the multi-epitope vaccine is shown in Figure 1.

### 2.1 HEV protein sequences

The amino acid sequences of different HEV proteins, including 961 ORF2 sequences and 746 ORF3 sequences of genotypes 1, 3, and 4, were retrieved from the National Center of Biotechnology Information protein database.

### 2.2 T-cell epitope prediction, including cytotoxic T-lymphocyte (CTL) epitopes and helper T-lymphocyte (HTL) epitopes

The Immune Epitope Database and Analysis Resource (IEDB)^1^ was used to predict the CD8 T-cell epitopes of the ORF2 and ORF3 proteins. NetMHCPan v4.1 was the prediction method used. Human was selected as the source species. A percentile rank of < 0.5 was considered the threshold (Reynisson et al., 2020).

Immune Epitope Database (IEDB) was also used to predict the MHC-II-binding (HTL) epitopes (15-mer) of the ORF2 and ORF3 proteins against human Human leukocyte Antigens (HLAs), such as HLA-DRB1*01:01, HLA-DRB1*03:01, HLA-DRB1*04:01, HLA-DRB1*04:05, HLA-DRB1*07:01, HLA-DRB1*08:02, HLA-DRB1*09:01, HLA-DRB1*11:01, HLA-DRB1*12:01, HLA-DRB1*13:02, and HLA-DRB1*15:01, with NN-align, SMM-align, CombLib, and Sturniolo. A percentile rank of < 2 was determined as the threshold.

### 2.3 B-cell epitope prediction

Three different methods were used to predict B-cell epitopes: the Emini surface accessibility scale, the Kolaskar and Tongaonkar (1990) antigenicity scale, and BepiPred v2.0 (Emini et al., 1985; Kolaskar and Tongaonkar, 1990; Closter et al., 2017). Duplicates in the epitopes predicted with the three methods were removed, and the remaining epitopes were used as candidate B-cell epitopes.

### 2.4 Assessment of screened epitopes for antigenicity, allergenicity, and toxicity

The antigenic potential of the T- and B-cell epitopes was predicted with VaxiJen v2.0, applying a threshold value of 0.5 (Doytchinova and Flower, 2007). The predicted T- and B-cell epitopes were then further evaluated in terms of their toxicity and allergenicity using the ToxinPred (Gupta et al., 2013) and AllergenFP v2.0 (Ivan et al., 2014) servers, respectively.

### 2.5 Construction of the polypeptide vaccine

Any multi-epitope vaccine constructed must be neither allergenic nor toxic. Good solubility of the vaccine when expressed at high levels must also be taken into account. Appropriate linkers, such as common rigid linker (EAAAK), fexible connecting peptide (GPGPG), The KK linker is a target sequence of the lysosomal protease histone B, which is one of the important proteases used for antigen processing in the context of MHC-II antigen presentation (KK), and AAY linkers act as cleavage sites for proteasomes in mammalian cells, contributing to the formation of natural epitopes and preventing the formation of “ligand epitopes”, thereby increasing the immunogenicity of epitope-based vaccines (AAY), were added to link the screened CTL, HTL and linear B-lymphocyte (LBL) epitopes. Human β-defensin 3 and LL37 were also added to the N-terminus of the vaccine sequence with the EAAAK linker, to increase the immunogenic capacity of the multi-epitope vaccine (Lande et al., 2021; Samad et al., 2022).

### 2.6 Immunogenic, allergenic, and physiochemical properties of the multi-peptide vaccine

The antigenicity of the multi-epitope vaccine polypeptide was predicted with the VaxiJen v2.0 tool, with a threshold value of 0.456. The allergenicity of the vaccine was analyzed with AllerTOP v.2.0 (Dimitrov et al., 2014). The ProtParam server was used to assess the physical and chemical properties of the construct (Majid and Andleeb, 2019), including the amino acid composition, molecular weight, theoretical isoelectric point (pI), grand average of hydropathicity (GRAVY), aliphatic and instability index, and *in vitro* and *in vivo* half-lives.

### 2.7 Vaccine structure modeling, refinement, and validation

The SOPMA server was used to predict the secondary structure of the polypeptide vaccine (Geourjon and Deléage, 1995). AlphaFold2 was used to model the tertiary structure of the vaccine. The structure was refined with the Galaxy server and validated with methods such as Ramachandran plots, and ProSA-web (Wiederstein and Sippl, 2007; Junsu et al., 2012; Wang et al., 2016).

### 2.8 Molecular docking and molecular dynamic (MD) simulation

The docking of the vaccine structure to TLR3 (PDB: 1ZIW) was performed by the HADDOCK server (van Zundert et al., 2016; Honorato et al., 2021). Molecular simulation was performed with GROMACS (version 2018.6) at 30 ns for 150,000 steps. Spc216.gro water was used as the solvent in which to simulate the proteins (Abraham et al., 2015). The addition of Na^+^ and Cl^–^ ions with the GROMACS tool Genion was used to neutralize the overall charge (Ikram et al., 2023). The energy of the protein structure was minimized in 50,000 steps and the protein was equilibrated with NVT isothermal–isochoric and NPT ensembles in 50,000 steps. MD simulation at 1500,000 steps was used.

### 2.9 Trial immune simulation *in silico*

Immune simulation was performed with the C-ImmSim server, which simulates the immune response with both systems’ biology techniques and information supported by data-driven predictive methods (Nicolas et al., 2010). The simulation was performed in 105 steps, and the default option was used for all HLA types of the host. Three different sets of injections were designed: virus alone, vaccine alone, and vaccine followed by virus. The shared sequence of the ORF2 and ORF3, respectively, was used as a substitute for the viral antigen sequence.

## 3 Results

### 3.1 T-cell and B-cell epitopes of HEV

The final screened CTL antigenic (MHC-I) epitopes, HTL (MHC-II) epitopes, and LBL epitopes in the ORF2 and ORF3 proteins of HEV are listed in Tables 1, 2. After they were checked with a series of server tools, the antigenic, nontoxic, and nonallergenic T-cell and B-cell epitopes that induce the cytokines interferon-gamma (IFN-γ), interleukin 4 (IL4), and IL10 were selected for the construction of the multi-epitope vaccine.

**TABLE 1:** Cytotoxic T-lymphocyte (CTL) epitopes and helper T-lymphocyte (HTL) epitopes in the ORF2 and ORF3 proteins selected for the polypeptide vaccine.

Protein	T-cell epitopes	Peptide	Allele	Antigenicity
ORF3	MHC-I	^53^AVPAVVSGV^61^	HLA-A*68:02/A*02:06/A*02:03/A*02:01	0.5575
ORF2	MHC-I	^277^RLHYRNQGW^285^	HLA-A*32:01	2.0994
ORF2	MHC-I	^204^TEASNYAQY^212^	HLA-B*44:02/B*44:03	0.6432
ORF2	MHC-I	^203^ATEASNYAQY^212^	HLA-A*01:01/B*44:03/B*44:02	0.6685
ORF2	MHC-I	^201^IMATEASNY^209^	HLA-B*15:01/A*30:02	0.7715
ORF2	MHC-I	^318^TPYTGALGL^326^	HLA-B*07:02	0.8121
ORF2	MHC-I	^220^RYRPLVPNA^228^	HLA-A*30:01	0.7326
ORF2	MHC-I	^325^GLLDFALEL^333^	HLA-A*02:03	1.2732
ORF2	MHC-I	^214^VVRATIRYR^222^	HLA-A*31:01	1.2896
ORF2	MHC-II	^155^RGA**ILRRQYNLS**TSPL^170^	HLA-DRB4*01:01	0.7185
ORF2	MHC-II	^217^AT**IRYRPLVPN**AV^229^	HLA-DRB1*09:01	1.09
ORF2	MHC-II	^323^ALG**LLDFALELE**^334^	HLA-DQA1*01:01/DQB1*05:01	1.6727
ORF2	MHC-II	^157^AI**LRRQYNLST**SPLT^171^	HLA-DRB1*04:01	0.7947
ORF2	MHC-II	^217^ATIRY**RPLVPNAVG**^230^	HLA-DRB1*09:01	1.1523
ORF2	MHC-II	^159^L**RRQYNLSTS**PLTS^172^	HLA-DRB1*09:01	0.7698
ORF2	MHC-II	^218^TIR**YRPLVPNAV**GGY^232^	HLA-DRB1*01:01	0.9212
ORF2	MHC-II	^212^**YRVVRATIR**YRPL^224^	HLA-DRB5*01:01/DPA1*02:01/DPB1*14:01	0.9773

The core peptides of the HTL epitopes are shown in bold. HLA naming principles, genetic loci * allele. *to separate genetic loci and allele.

**TABLE 2:** Linear B-lymphocyte (LBL) epitopes in the ORF2 and ORF3 proteins selected for the polypeptide vaccine.

Protein	B-cell epitopes	Peptide	Antigenicity
ORF3	B	^69^PSPSPI^74^	0.983
ORF2	B	^469^DYDNQHEQDRPTPSPAPSRPF^489^	0.7828
ORF2	B	^323^ALGLLDFALEL^333^	1.0275
ORF2	B	^160^RRQYNLS^166^	0.9911
ORF2	B	^444^ENAQQD^449^	1.1613
ORF2	B	^469^DYDNQHEQDRPTPSPAPSR^487^	0.9301

### 3.2 Construction of the multi-epitope vaccine polypeptide

In total, nine CTL, eight HTL, and six LBL epitopic peptides were fused together with the GPGPG, KK, and AAY linkers, respectively, to create the multi-epitope vaccine construct. To boost the immunogenicity of the construct, human β-defensin 3 (GII NTLQKYYCRVRGGRCAVLSCLPKEEQIGKCSTRGRKCCRRKK) and LL37 (LLGDFFRKSKEKIGKEFKRIVQRIKDFLRNLVPRTES) were added as adjuvants to the amino terminus of the polypeptide, joined to the first CTL epitope with the EAAAK linker. An additional start codon was added at the start of the sequence. The primary structure of the multi-epitope subunit vaccine construct included a total of 433 amino acids. The sequence is as follows: MGIINTLQKYYCRVRGGRCAVLSCLPKEEQIGKCSTRGRKCCR RKKEAAAKLLGDFFRKSKEKIGKEFKRIVQRIKDFLRNLVPRTE SEAAAKAVPAVVSGVGPGPGRLHYRNQGWGPGPGTEASNYA QYGPGPGATEASNYAQYGPGPGIMATEASNYGPGPGTPYTG ALGLGPGPGRYRPLVPNAGPGPGGLLDFALELGPGPGVVRATI RYRGPGPGRGAILRRQYNLSTSPLKKATIRYRPLVPNAVKKAL GLLDFALELEKKAILRRQYNLSTSPLTKKATIRYRPLVPNAVGK KLRRQYNLSTSPLTSKKTIRYRPLVPNAVGGYKKYRVVRATIRY RPLKKPSPSPIAAYDYDNQHEQDRPTPSPAPSRPFAAYALGLLD FALELAAYRRQYNLSAAYENAQQDAAYDYDNQHEQDRPTPS PAPSR.

### 3.3 Immunogenicity, allergenicity, and physiochemical properties of the vaccine candidate

The assessment of the immunogenic, allergenic and solubility characteristics of the construct suggested that the multi-epitope vaccine designed here was a priority (Table 3). The calculated molecular weight was 47.4 kDa and the pI 10.20, indicating the basic nature of the vaccine. The instability index, 42.80, indicated its good stability. The GRAVY value was −0.636, indicating its hydrophilic characteristics, which allows it to connect with other proteins. The aliphatic index of the peptide was 71.76, indicating the high thermostability of the vaccine. The estimated half-life was 30 h in mammalian reticulocytes *in vitro*, and > 20 h in yeast *in vivo*, and > 10 h in *E. coli in vivo*.

**TABLE 3:** Immunogenicity, allergenicity, and physiochemical properties of the vaccine.

Features	Assessment
Number of amino acids	433
Molecular weight	47.4 kDa
Isoelectric point	10.20
Instability index	42.80
Aliphatic index	71.76
Grand average of hydropathicity (GRAVY)	−0.636

### 3.4 Secondary structure and three-dimensional (3D) structure of multi-epitope vaccine

**FIGURE 2:**
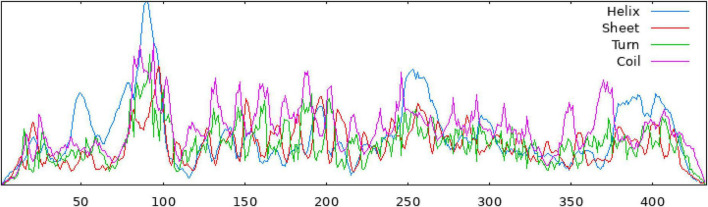
The secondary structure of the multi-epitope vaccine, as predicted by the SOPMA server.

**FIGURE 3:**
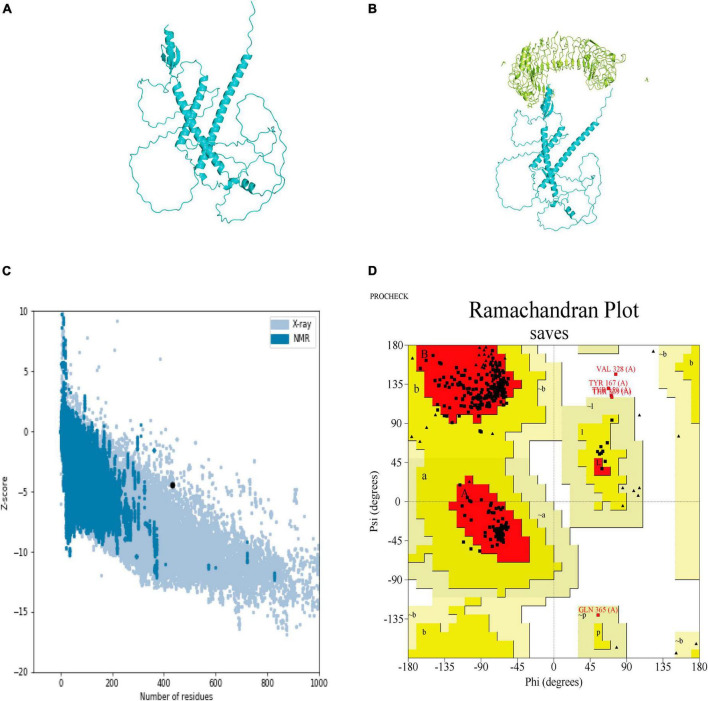
Predicted 3D structure of the multi-epitope vaccine and its validation with Pro-SA and Ramachandran plots. **(A)** Predicted structure of multi-epitope vaccine. **(B)** TLR3-docked structure with a HADDOCK score of –60.97 and a Z-score of –2.4. **(C)** Pro-SA with a Z-score of –4.39. **(D)** Ramachandran plots with 93.2% of residues in most-favored regions.

The secondary structure of the construct showed that the polypeptide contained 49.65% coil, 31.18% helix, 16.40% strand, and 2.77% beta turn (Figure 2). The sequence of the multi-epitope vaccine was submitted to AlphaFold2. AlphaFold2 uses the predicted local distance difference test loss (pLDDT) to directly derive the per-position confidence of predicted models (Figures 3A, B). The pLDDT score of this model was 75.45. The 3D structure was confirmed with Pro-SA with a Z-score of −4.39, indicating the good quality of the vaccine. On Ramachandran plots, the percentage of residues in the most-favored regions was 93.2%, and only 1.2% residues were in the region that was not allowed. Taken together, these parameters indicate the high quality of the multi-peptide vaccine (Figures 3C, D).

### 3.5 Docking the vaccine structure to TLR3 and molecular dynamic (MD) simulation

HADDOCK clustered 160 structures in 11 clusters, which represented 80% of the water-refined models generated by HADDOCK. The best structure of the top 10 clusters was selected according to its HADDOCK score of −60.97 and Z-score of −2.4 (Figure 3B). The more negative the Z-score, the more optimal the structure.

**FIGURE 4:**
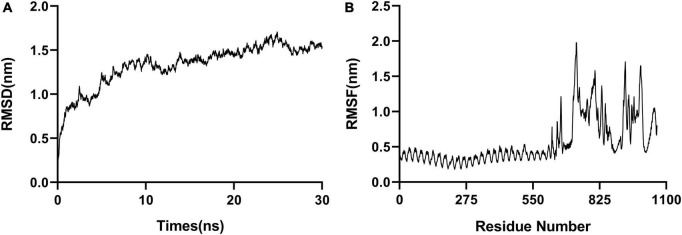
Molecular dynamic simulations. **(A)** RMSD, with an average value of 1.3 nm. **(B)** RMSF, with a mean value of 0.6 nm.

Molecular dynamic (MD) simulation of the complex formed between the polypeptide vaccine and TLR3 was performed. The stability of the docked complex was reflected in the root mean square deviation (RMSD), which had an average value 1.3 nm (Figure 4A). The root mean square fluctuation (RMSF) was between 0.2 and 2.0 nm, with a mean value 0.6 nm (Figure 4B), reflecting the good stability of the whole complex. The high-fluctuation residues (including R666, G730, G807, R931, D995, and Q1051) were distributed in the flexible loop area.

### 3.6 Simulated immunization with C-ImmSim

**FIGURE 5:**
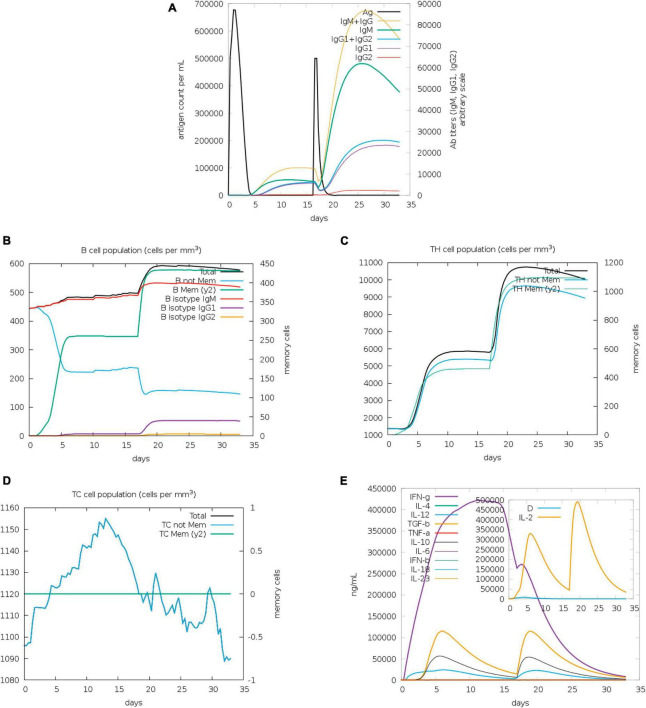
Immunization simulated with C-ImmSim. **(A)** The virus, immunoglobulins, and immunocomplexes. IgM and IgG levels clearly increased, whereas IgG2 levels increased less obviously. **(B)** B memory (y2) and B-isotype IgM levels increased. **(C,D)** TH memory (y2), TC, and TH cells generally showed an increase. **(E)** IFN-γ levels increased notably after stimulation.

The assessment of antibody levels provides crucial insights into the efficacy of vaccines and the immune response to pathogens. The role of vaccines in influencing the immune response to infection can be clarified by examining the dynamics of CTL and HTL populations. Our results showed increased immunoglobulin M (IgM) and IgG after 20 days of stimulation with antigen, whereas increases of IgG2 were less obvious (Figure 5A). B memory (y2) and B-isotype IgM levels were elevated (Figure 5B). In general, the levels of TH memory (y2), TC, and TH cells were also increased (Figures 5C, D). The IFN-γ level peaked after 10 days of stimulation (Figure 5E).

## 4 Discussion

Hepatitis E virus (HEV) is a zoonotic virus that is transmitted through fecal and oral transmission. There is currently no universal vaccine available for HEV (Dalton et al., 2008; Nan and Zhang, 2016). Traditional vaccines targeting ORF2 have been developed for many years (Zhu et al., 2010; Zhang et al., 2015). Multi-epitope vaccines are an emerging area of research interest. An advantage of multi-epitope vaccines is that they reduce adverse reactions (i.e., allergy) and the antigenic load, eliciting a more specific immune response to conserved epitopes (Oli et al., 2020). A combination of the dominant epitopes of different antigens is superior to a single antigen epitope at inducing an immune response (Samad et al., 2022). Multi-epitope vaccines have been used to inhibit a variety of pathogens. For example, multi-epitope vaccines targeting Epstein–Barr virus (EBV) and influenza virus successfully elicit robust neutralizing antibodies and T-cell responses. The EBV polyepitope vaccine, which includes 20 CD8^+^ T-cell epitopes, induces high frequencies of CD4^+^ T cells and CD8^+^ T cells, which are maintained for more than 7 months (Dasari et al., 2023). It has been reported that the norovirus (NoV) P particle, a 24-self-assembly of the protruding domain, displays H16, M2e, and NP9 on its surface, which elicit long-lasting hemagglutinin (HA)-specific neutralizing antibodies, M2e-CD4^+^ and CD8^+^ T-cell responses, and a nucleoprotein-specific CTL response, respectively (Nie et al., 2023).

The HEV exists in two states, quasi-enveloped virions, which have a surface membrane and non-enveloped naked virions. The ORF3 and ORF2 antigens are located on the surface of these two types of virions, respectively (Takahashi et al., 2008). Considering the reduction in antibody-mediated neutralization of HEV resulting in antibody evasion by the secreted form of ORF2 (Yin et al., 2018) and the partly protective ORF3 presented on the surface of quasi-enveloped HEV (Takahashi et al., 2008), we first induced ORF3 as an epitopic candidate combined with ORF2 as a multi-peptide vaccine. As previous studies have demonstrated, ORF3 can partially neutralize quasi-enveloped HEV (Ma et al., 2009; Syed et al., 2017; Maurer et al., 2022). The designed vaccine will therefore potentially possess efficacy against both non-enveloped and quasi-enveloped HEV.

In this study, we constructed peptide epitopic vaccines targeting HEV protein ORF2 and ORF3 through computational biology. We screened 9 CTL antigenic (MHC-I) epitopes, 8 HTL (MHC-II) epitopes, and 6 LBL epitopes that covered a wide population of up to 90.60%. On evaluation, these antigens showed good immunogenicity, low allergenicity and non-toxicity. The multi-epitope vaccine is connected through GPGPG, KK, AAY, and EAAAK connectors, and human β-defensin 3 and LL37 are used as adjuvants to enhance the immune response. As the 3D structure validated by Ramachandran plots, the percentage of residues in the most-favored regions was up to 93.2%, and only 1.2% of residues were disallowed. This is in accordance with the good quality model for Ramachandran plots, which is over 90% in the most favored regions (Hooft et al., 1997). Our multi-epitopic vaccine has potential efficacy and low allergenicity against HEV. The Z-score of the 3D structure docked with TLR3 was −2.4 by HADDOCK. The more negative the Z-score, the better the docked structure. This score suggests that the docked structure is of good quality. It has been reported that abundant TLR3 and IFNγ levels were helpful to inhibit HEV and promote recovery (Majumdar et al., 2015; Li et al., 2019), it appears likely that our design will lead to a promising result. Under simulated immunization with the peptide vaccine, both IgM and IgG levels were significantly elevated, as were the abundance of B cells, T cells, and IFN-γ. It suggests the multi-peptide vaccine will induce robust cellular and humoral immune responses effectively. These epitopes may provide a reference for the design of other ORF2 and ORF3 vaccines in the future.

The epitopes that cause excellent immune responses are mainly concentrated on ORF2 and ORF3 (Dawson et al., 1992; Khudyakov et al., 1993; Riddell et al., 2000; Srivastava et al., 2007; Coursaget et al., 2010; Favorov et al., 2010) and the existing HEV epitopic vaccines mainly target ORF2. In these studies, the parameter settings like the selected HLA range and the server threshold settings determine the final peptide selection. The selected peptides all differ between vaccine studies but effective immunoreactions have been induced using these different strategies (Samavia et al., 2022; Anwar et al., 2023; Ikram et al., 2023). The findings of our study are novel in that ORF3 was included as an additional target along with ORF2.

## 5 Conclusion

Hepatitis E is a self-limiting disease, but presents a serious threat to pregnant women and immunocompromised individuals (Qian et al., 2023). There is no global vaccine currently available against HEV. Using an immunoinformatic approach, we designed a polypeptide vaccine against the ORF2 and ORF3 proteins of HEV, which was capable of inducing strong T- and B-cell responses by simulated immunization. This is the first study to consider ORF3 as a multi-epitope candidate to combat HEV. Our design provides potential efficacy against both non-enveloped and quasi-enveloped HEV, however, the efficacy of this vaccine requires experimental validation.

## Data availability statement

The raw data supporting the conclusions of this article will be made available by the authors, without undue reservation.

## Author contributions

QL: Data curation, Formal analysis, Methodology, Software, Writing – original draft, Writing – review & editing. HW: Data curation, Methodology, Software, Writing – review & editing. JM: Formal analysis, Writing – review & editing. JWa: Formal analysis, Methodology, Software, Writing – original draft. JWu: Formal analysis, Writing – review & editing. SL: Formal analysis, Writing – review & editing. JT: Formal analysis, Writing – review & editing. JN: Project administration, Writing – review & editing. WH: Project administration, Writing – review & editing.

## Footnotes

1
http://tools.iedb.org/mhci
